# Robust Prediction of Anti-Cancer Drug Sensitivity and Sensitivity-Specific Biomarker

**DOI:** 10.1371/journal.pone.0108990

**Published:** 2014-10-17

**Authors:** Heewon Park, Teppei Shimamura, Satoru Miyano, Seiya Imoto

**Affiliations:** Human Genome Center, Institute of Medical Science, University of Tokyo, Tokyo, Japan; University of Granada - Q1818002F, Spain

## Abstract

The personal genomics era has attracted a large amount of attention for anti-cancer therapy by patient-specific analysis. Patient-specific analysis enables discovery of individual genomic characteristics for each patient, and thus we can effectively predict individual genetic risk of disease and perform personalized anti-cancer therapy. Although the existing methods for patient-specific analysis have successfully uncovered crucial biomarkers, their performance takes a sudden turn for the worst in the presence of outliers, since the methods are based on non-robust manners. In practice, clinical and genomic alterations datasets usually contain outliers from various sources (e.g., experiment error, coding error, etc.) and the outliers may significantly affect the result of patient-specific analysis. We propose a robust methodology for patient-specific analysis in line with the NetwrokProfiler. In the proposed method, outliers in high dimensional gene expression levels and drug response datasets are simultaneously controlled by robust Mahalanobis distance in robust principal component space. Thus, we can effectively perform for predicting anti-cancer drug sensitivity and identifying sensitivity-specific biomarkers for individual patients. We observe through Monte Carlo simulations that the proposed robust method produces outstanding performances for predicting response variable in the presence of outliers. We also apply the proposed methodology to the Sanger dataset in order to uncover cancer biomarkers and predict anti-cancer drug sensitivity, and show the effectiveness of our method.

## Introduction

Recently, numerous studies have attempted to personalized therapy and medicine based on advanced biomedical technologies [2], [9]. A crucial issue for personal genome research is to reveal the genomic features of an individual patient that are relevant for treatment. The elastic net-type regularized regression (e.g., ridge [11], lasso [29], elastic net [34], etc.) has been widely used to uncover biomarkers, and successfully performed for identifying genomic features and predicting response variable based on high-dimensional gene expression dataset. The methods, however, can only provide results based on the average genomic features of all patients. In essence, it is not yet possible to use these methods to identify genomic features for an individual patient, thus it is hard to effective personalized treatment and medicine.

Wang et al. [30] considered the patient-specific pathway activities based on a mixed model, where the fixed effects modeled the mean pathway of gene expressions profiles for patient groups and random effects described patient variations from the group mean. Shimamura et al. [28] proposed a method, called a NetworkProfiler, for identifying patient-specific gene regulatory networks based on a varying coefficient model and kernel-based elastic net-type regularized regression. By using a Gaussian kernel function, the NetworkProfiler can effectively perform patient-specific analysis based on neighborhood samples around a patient. Although the existing elastic net-type regularization methods perform effectively for patient specific analysis, their performances take a sudden turn for the worst in the presence of outliers, because the methods are constructed by non-robust manners (e.g., least square loss function). In practice, the clinical and genomic alterations datasets usually contain outliers from various sources (e.g., experiment error, coding error, etc.), and thus the existing methods cannot effectively uncover patient-specific biomarkers and predict anti-cancer drug sensitivity.

Although the issue is critically important, relatively little attention has been paid to the robustness of patient-specific analysis. We consider a robust method to uncover patient-specific genomic features and predict anti-cancer drug response in line with the NetworkProfiler. The genomic alterations dataset is usually constructed with a large number of features for a small number of samples (i.e., high dimensional dataset), and detecting and controlling outliers in a high dimensional dataset are difficult tasks. We refer to the method for controlling outliers by using the robust Mahalanobis distance based on principal component analysis (PCA) [25]. By using the principal components, we can detect outliers in a high dimensional genomic alteration dataset based on robust Mahalanobis distance by overcoming calculation of inverse covariance matrix. Furthermore, because the principal component space is defined by maximize the variance along each component, and outliers increase the variance of the data, we can effectively perform outlier detection [5], 25.

We propose a robust modeling strategy for patient-specific analysis, which infers patient-specific biomarkers associated with anti-cancer drug response. The proposed strategy is based on kernel-based elastic net-type regularization, and thus can perform patient-specific analysis through neighborhood samples around a target patient. Furthermore, our method can perform effectively for predicting anti-cancer drug sensitivity and identifying drug response-specific biomarkers for each patient even in the presence of outliers, since the method is based on a robust regularized regression by using a weight through the Mahalanobis distance in principal component space [25].

We conduct Monte Carlo simulations to examine the effectiveness of the proposed method, and show the outstanding performance of our method in the view point of prediction accuracy. We also apply the proposed modeling strategy to the publicly available Sanger Genomic of Drug Sensitivity in Cancer dataset from the Cancer Genome Project (http://www.cancerrxgene.org/). Our methodology uncovers biomarkers for individual patients and predicts anti-cancer drug response given as IC50 values based on gene expression levels. Though Monte Carlo simulations and application to the Sanger dataset, we can see that our method performs effectively for patient-specific feature selection and prediction of interesting response variable, even in the presence of outliers.

## Methods

Suppose we have 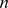 independent observations 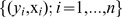, where 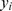 are random response variables (e.g., anti-cancer drug response) and 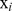 are *p*-dimensional vectors of the predictor variables (e.g., genomic alterations). Consider the linear regression model, 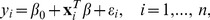
(1)where 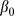 is an intercept, 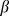 is an unknown *p*-dimensional vector of regression coefficients and 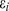 are the random errors that are assumed to be independently and identically distributed with mean 0 and variance 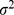.

To uncover a biomarker, the elastic net-type regularization methods (e.g., ridge, lasso, elastic net, etc.) have been widely applied, and used successfully to identify crucial genes based on the following optimization problem, 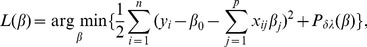
(2)where 
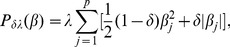
(3)and where 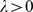 is a regularization parameter controlling model complexity. The penalty term of the elastic net is a convex combination of the ridge and lasso penalties. When 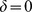, the elastic net becomes the ridge regression with a 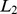 penalty, whereas when 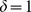, it becomes the lasso with a 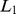 penalty. The elastic net performs variable selection and estimation along with the properties of both lasso and ridge regression for 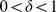.

The elastic net-type penalties enable us to simultaneously identify crucial biomarkers and predict drug response. Furthermore, we can effectively perform regression modeling in the high dimensional genomic alterations dataset and in the presence of multicollinearity by imposing the penalty on the least squares loss function. Although the existing methodologies successfully identify crucial biomarkers and show remarkable performance for predicting drug response, they have been used to identify averaged biomarkers for all patients. In other words, the existing method cannot identify patient-specific characteristics in a disease.

### NetworkProfiler

Shimamura et al. [28] proposed a novel statistical method for inferring patient-specific gene regulatory networks based on a varying-coefficient structural equation model. Let 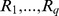 be 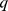 possible regulators, and 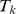 be the 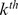 target gene controlled by the 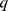 regulators at 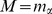 [28]. The varying coefficient structural equation model for 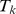 is given as 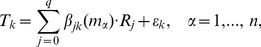
(4)where 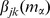 is a regression coefficient of 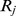 on 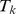 for the modulator 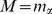. The patient-specific regression coefficients 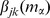 are estimated via the kernel-based regularization method by minimizing, 
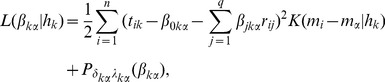
(5)where 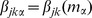, and 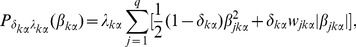
(6)where 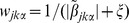 is a weight for a recursive elastic net penalty for effective feature selection [28], and 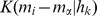 is a Gaussian kernel function with bandwidth 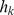, 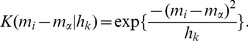
(7)


The Gaussian kernel function 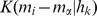 is used to fit the model at 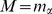 based on samples in the neighborhood around the 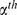 patient. By using the Gaussian kernel function in regularized regression, the NetworkProfiler performs effectively to infer patient-specific gene regulatory networks, and the results enable us to effective personalized anti-cancer therapy.

It is, however, well known that the genomic alterations datasets usually contain outliers from various sources (e.g., experiment error, coding error, etc.). It implies that the existing method would not perform well for uncovering biomarkers and predicting anti-cancer drug response, because the existing method in (5) is based on a penalized least squares loss function. It was previously shown that the elastic net-type regularization methods that are based on least square loss function perform poorly in the presence of outliers, and several robust methodologies have been proposed to overcome the drawbacks of a least square loss function in regularized regression modeling [1], [14], [25].

We propose a robust method for patient-specific analysis in line with the NetworkProfiler.

### Robust regularization for outlier-resistant patient-specific analysis

**Figure 1:**
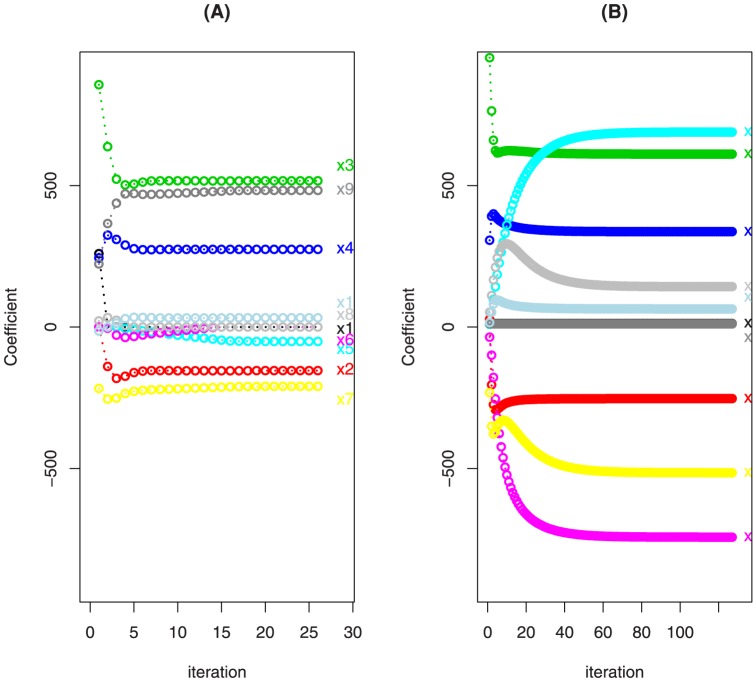
Iteration for coefficients in the regularized regression modeling with lasso (i.e., *δ* = 1) penalty.

We first show how outliers could affect the estimation process when using the penalized least squares methodology. Figure 1 shows the iteration for coefficients during optimization of the regularized regression modeling with a lasso penalty [25] under the original and contaminated diabetes datasets [3] in (A) and (B), respectively. The contaminated dataset contains 10% outliers for 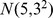 in 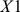 and 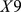 among the 10 predictor variables. The coefficients converged after 26 iterations in the original dataset, as shown in Figure 1 (A). In the presence of outliers, however, the optimization procedure with the lasso estimator is disturbed and the iteration number required for convergence is significantly increased as shown in Figure 1 (B). This implies that outliers significantly disturb the regularized regression modeling, and thus may lead to poor results in uncovering biomarkers and predicting drug response where patient-specific analysis.

We propose a robust method to effectively uncover patient-specific cancer biomarkers and predict anti-cancer drug sensitivity in line with the NetworkProfiler. The genomic features dataset is constructed with a large number of features and a relatively small number of samples (i.e., high dimensional dataset), and detecting and controlling outliers in a high dimensional dataset are generally difficult tasks. To resolve the issue, we consider the weight for controlling outliers based on robust Mahalanobis distance calculated in robust principal component space, as previously demonstrated by Park and Konishi [25], 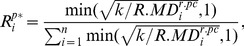
(8)where 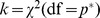 is the 95% quantile of the 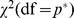 distribution [14], and 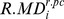 is a robust Mahalanobis distance based on the robustly estimated mean 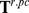 and covariance matrix 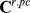 by Minimum Volume Ellipsoid (MVE) calculated in the robust principal components 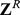 space as follows, 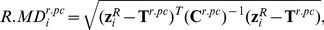
(9)where 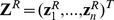 is a 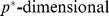 matrix of robust principal components based on robust loadings by using the projection-pursuit technique [12]. By using the robust principal component space, we can effectively detect outliers based on the robust Mahalanobis distance, thereby overcoming the calculation of the inverse covariance matrix in a high dimensional dataset. Furthermore, the principal components space is defined by maximizing the variance along each component, and since outliers increase the variance of dataset, we are able to more faithfully detect outliers [5]. It implies that the weight 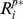 based on the robust Mahalanobis distance calculated in robust principal component space is a useful tool for controlling outliers in high dimensional genomic data.

We refer to the weight in (8) for outlier-resistant patient-specific analysis, and propose a robust method for uncovering biomarkers and predicting drug sensitivity for an individual patient as follows, 
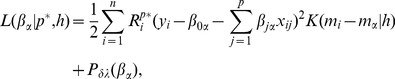
(10)

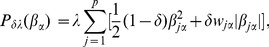
(11)where 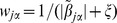 is a weight of the adaptive elastic net penalty [35].

The proposed modeling strategy is effectively conducted by using the coordinate descent algorithm based on the weighted update [6]. Our method can efficiently perform patient-specific analysis based on the Gaussian kernel function, and its effective performance can be consistently provided even in the presence of outliers by controlling outliers through the weight.

## Results

We examine the effectiveness of the proposed modeling strategy as a robust method for patient-specific analysis through Monte Carlo simulations and application to cancer genomics data. To evaluate the proposed method, we compare the prediction accuracy and variable selection results of our method, the NetworkProfiler and elastic net. In our study, the NetworkProfiler is used to uncover individual biomarkers instead of gene networks. For the numerical studies, we use the adaptive elastic net penalty 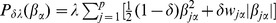 [35] in the proposed method, NetworkProfiler and elastic net. We choose the tuning parameters 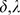 and bandwidth 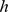 in Gaussian kernel function based on k-fold cross validation [18], 
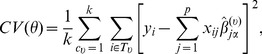
(12)where 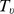 is 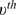 validation samples for 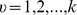, and the data 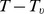 is used to estimate for each 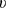. In numerical studies, we use the 3-fold cross validation, which has been used in high dimensional data analysis like genomic data analysis [13], [20], [22], [26], [32]. The robust Mahalanobis distance is calculated based on the robust principal components that contributed 95% of the total variation.

### Monte Carlo simulations

We simulated 100 datasets consisting of 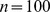 observations from the model 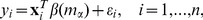
(13)where 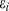 are assumed to be distributed as 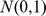 and 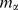 are generated from a uniform distribution 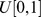 for 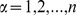. The correlation between 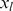 and 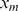 is 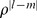 with 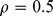 in 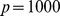 dimensional multivariate normal distribution with mean zero. We consider a 1000 dimensional vector of coefficients with randomly selected 100 non-zero and 900 zero coefficients.

**Figure 2:**
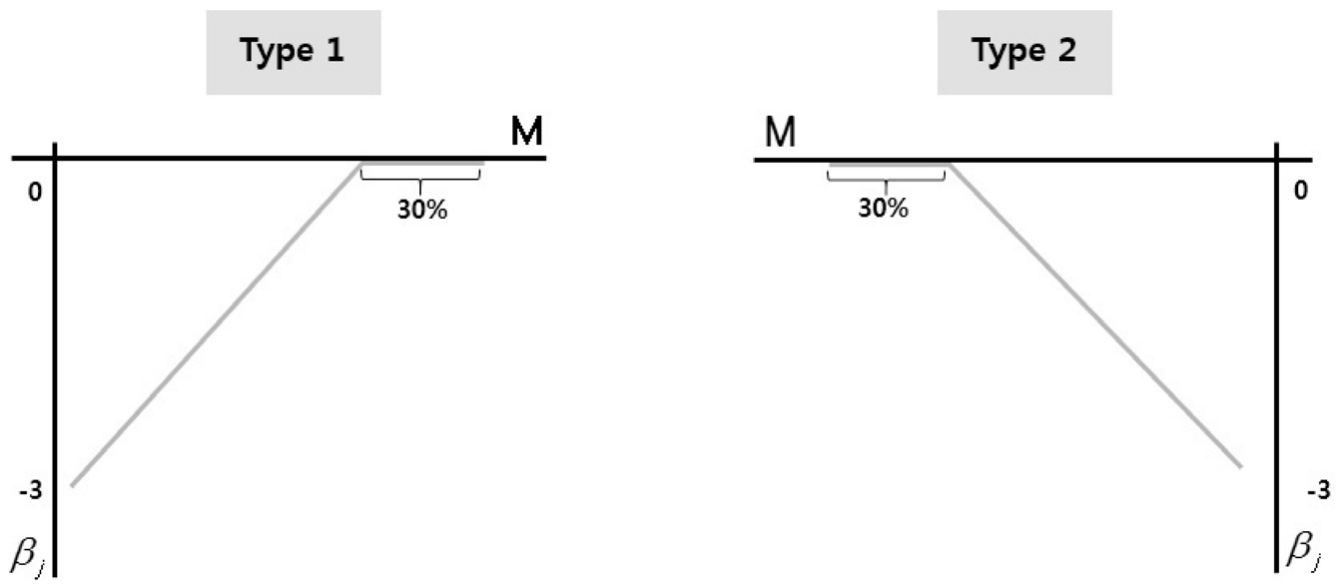
Coefficient functions of varying coefficient model.

Two types of coefficient functions in the above varying coefficient model are considered, as shown in Figure 2. We consider 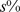 of samples as outliers in 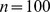 samples. If the 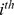 sample is an outliers, 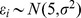 and 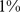 of 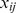 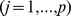 follow 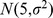. Here we set 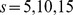, and 20, and 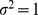 and 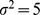 in simulations 1 and 2, respectively.

We consider a training dataset with 75 samples and a test dataset with 25 samples in each 100 simulations. The hyperparameters are selected by 3-fold cross validation in the training dataset, and the prediction errors are calculated in test dataset based on the selected parameters. We then compare the prediction accuracy given as average of the median squared error, and the variable selection accuracy given as true positive (i.e., average percentage of non-zero coefficients, that were estimated as non-zero) and true negative (i.e., the average percentage of true zero coefficients, that were correctly set to zero) for each of the 100 generated datasets.

A large number of predictor variables leads to time consuming analysis, and thus increases the overall computational cost of a method. Furthermore, it has been exposed that a large number of predictor variables with noisy genes may disturb the modeling procedure, and thus leads to poor prediction results [19]. Table 1 shows the prediction accuracy of the NetworkProfiler based on all features and based on a pre-selected small number of features that have the highest variance. It can be seen through Table 1 that consideration of all features does not produce high prediction performance compared with the performance of a regression model built on a pre-selected small number of features. It implies that there is no need to consider all features for patient-specific analysis, because it leads to inefficient modeling without improving model performance.

**Table 1:** Comparison prediction accuracy of model with *p* = 1000 and *p* = 200.

	5%		10%		15%		20%	
	*N*(5, 5)	*N*(5, 1)	*N*(5, 5)	*N*(5, 1)	*N*(5, 5)	*N*(5, 1)	*N*(5, 5)	*N*(5, 1)
*p*1000	**0.259**	0.333	0.266	**0.254**	0.259	0.289	0.251	0.274
*P*200	0.280	**0.290**	0.266	0.290	**0.227**	**0.288**	0.251	**0.254**

Thus, we compare the proposed robust method to the NetworkProfiler and elastic net based on model with 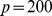 predictor variables that have the highest variance in all samples. Tables 2 and 3 show the simulation results (i.e., true positive (T.P), true negative (T.N) and prediction error (P.E)) in simulations 1 and 2, respectively, where the bold values indicate the best performance among the three methods (i.e., elastic net: ELA, NetworkProfiler: NP, robust method: R). The varying coefficient model produces discriminative variable selection results in each sample, and thus we only compare the feature selection results of the NetworkProfiler and proposed robust one, because the elastic net cannot perform sample-specific feature selection.

**Table 2:** Results of simulation 1 with Outlier for *N*(5, 1).

		Type 1			Type 2		
		T.P	T.N	P.E	T.P	T.N	P.E
	ELA	-	-	0.338	-	-	0.324
5%	NP	0.71	1.00	0.290	0.70	1.00	0.276
	R	0.71	1.00	**0.285**	0.70	1.00	**0.271**
	ELA	-	-	0.325	-	-	0.329
10%	NP	0.69	1.00	0.290	0.70	1.00	0.310
	R	0.69	1.00	**0.284**	0.70	1.00	**0.303**
	ELA	-	-	0.289	-	-	0.294
15%	NP	0.71	1.00	0.288	0.70	1.00	0.264
	R	0.71	1.00	**0.287**	0.70	1.00	**0.258**
	ELA	-	-	0.285	-	-	0.259
20%	NP	0.71	1.00	0.254	0.69	1.00	0.258
	R	0.71	1.00	**0.244**	0.69	1.00	**0.255**

**Table 3:** Results of simulation 2 with Outlier for *N*(5, 5).

		Type 1			Type 2		
		T.P	T.N	P.E	T.P	T.N	P.E
	ELA	-	-	0.321	-	-	0.314
5%	NP	0.69	1.00	0.280	0.70	1.00	0.277
	R	0.69	1.00	**0.278**	0.70	1.00	**0.271**
	ELA	-	-	0.298	-	-	0.280
10%	NP	0.70	1.00	0.266	0.70	1.00	0.251
	R	0.70	1.00	**0.262**	0.70	1.00	**0.249**
	ELA	-	-0	0.261	-	-0	0.255
15%	NP	0.71	1.00	0.227	0.69	1.00	0.240
	R	0.71	1.00	**0.225**	0.69	1.00	**0.231**
	ELA	-	-	0.290	-	-	0.229
20%	NP	0.71	1.00	0.251	0.70	1.00	0.214
	R	0.71	1.00	**0.249**	0.70	1.00	**0.211**

Tables 2 and 3 show that the proposed robust method for patient-specific analysis outperforms for predicting response variable in all simulation situations and coefficient function types. We also observe that the proposed robust method and NetworkProfiler make no difference results in variable selection. From the results, we can see that controlling outliers in the modeling procedure produces outlier-resistant estimation results, and the results lead to outstanding prediction of interesting response variable.

### Real world example: Sanger dataset

We apply the proposed modeling strategy to the publicly available Sanger Genomics of Drug Sensitivity in Cancer dataset from the Cancer Genome Project (http://www.cancerrxgene.org/). The main goal of the project is to identify the molecular features of various cancers and to predict sensitivity of anti-cancer drugs. The dataset consists of gene expression levels, copy number and mutation status for 654 cell lines. The IC50 values (i.e., half maximal inhibitory drug concentrations) of 138 drugs are given as the natural log of drug sensitivity value. The IC50 values from the Sanger dataset contain not a few of missing values, and thus we perform biomarkers discovery and anti-cancer drug response prediction based on 200 randomly selected samples, of which 150 cell lines were used as a training data and 50 cell lines were used as a test data for each of the 138 drugs.

**Figure 3:**
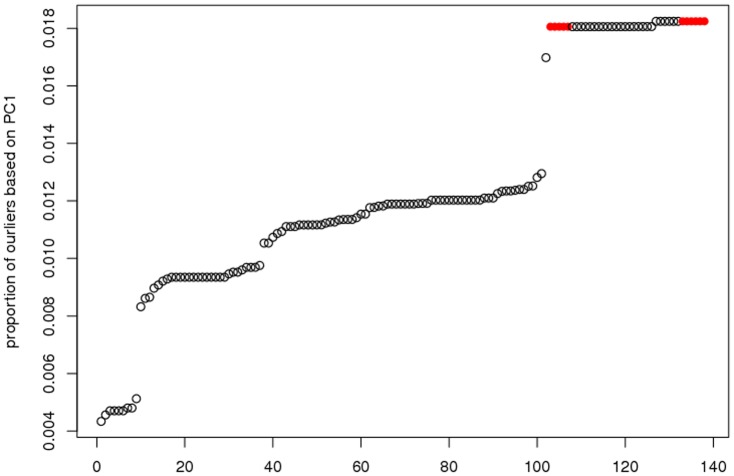
Sorted *C^dr^* values of 138 dataset.

To evaluate the proposed robust methodology, we first decide whether the dataset constructed with IC50 values of each drug and expression levels of 13,321 genes is contaminated or not. For each of the 138 dataset (i.e., gene expression levels and IC50 values) corresponding 138 drugs, we find a first principal component of the dataset, and then decide based on the following criterion, 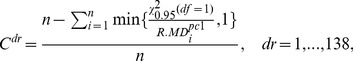
(14)where 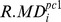 is the robust Mahalanobis distance calculated from the first principal component. The criterion 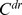 has a zero value in a non-contaminated dataset, while a large value of 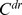 indicates that the dataset contains outliers. Figure 3 shows the sorted 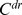 values for the 138 datasets.

We consider the datasets with 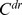 located in top-right side of Figure 3 as contaminated datasets, which have relatively large 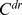 values that are highly deviated from the mean of 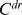 values. The proposed robust method is then applied to the contaminated datasets to evaluate the performance of the methods when identifying biomarkers and predicting anti-cancer drug sensitivity. We compare the prediction accuracy based on 10 datasets corresponding to the 10 drugs shown as red dots in Figure 3: drugs FTI.277, DMOG, NSC.87877, AKT.inhibitor.VIII, Midostaurin, BMS.754807, Thapsigargin, Bleomycin, Doxorubicin, Epothilone.B.

As mentioned previously, a large number of features not only leads to inefficient modeling, but may also produce poor results compared with modeling based on a pre-selected small number of features. We first compare the prediction accuracy (i.e., median squared error of 50 test samples) of anti-cancer drug response based on expression levels of 133 (1% of total 13,321 genes) genes and the 500 genes that have the highest variance based on the NetworkProfiler in Table 4. Table 4 shows that modeling based on the expression levels of 133 genes produces outstanding prediction accuracy compared with modeling based on 500 genes. From the result, we can also conclude that there is no need to consider a large number of genes with noise, and that a large number of features only leads to inefficient modeling and poor prediction results. Thus, we evaluate the proposed robust method compared with the NetworkProfiler and elastic net based on the expression levels of 133 genes. Table 5 shows the median squared error of 50 test samples as a prediction error of anti-cancer drug response. The proposed robust method outperforms the existing methods for predicting anti-cancer drug response in the contaminated datasets.

**Table 4:** Prediction results of drug sensitivity by using NetworkProfiler based on 133 and 500 genes.

	FTI.277	DMOG	NSC.87877	AKT.inhibitor.VIII	Midostaurin
p500	0.402	**0.222**	0.208	0.303	0.263
p133	**0.291**	0.239	0.211	**0.232**	**0.134**

	BMS.754807	Thapsigargin	Bleomycin	Doxorubicin	Epothilone.B
p500	**0.107**	0.140	0.201	0.318	0.760
p133	0.124	**0.131**	**0.049**	**0.182**	**0.725**

**Table 5:** Comparison of prediction accuracy of drug sensitivity.

	FTI.277	DMOG	NSC.87877	AKT.inhibitor.VIII	Midostaurin
R	0.293	**0.220**	**0.162**	**0.177**	**0.120**
NP	0.291	0.239	0.211	0.232	0.134
Elastic net	**0.269**	0.561	0.323	0.447	0.477

	BMS.754807	Thapsigargin	Bleomycin	Doxorubicin	Epothilone.B
R	**0.099**	**0.120**	**0.044**	**0.153**	**0.621**
NP	0.124	0.131	0.049	0.182	0.725
Elastic net	0.720	0.274	0.279	0.367	0.954

**Figure 4:**
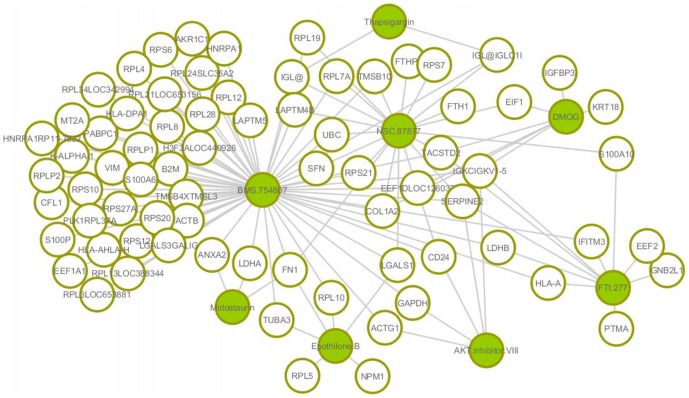
Identified biomarkers on each anti-cancer drug.

Figure 4 shows the uncovered cancer biomarkers that are selected in greater than 80% of models for the each 150 tissues (i.e., selected in greater than 120 samples based on varying coefficient model) by our method for each drug's response. In order to show a reliability of our method, we also show the 10 most frequently discovered genes when predicting the sensitivity of 10 drugs and their references in Table 6. There are differences between the biomarkers discovered based on our method and those discovered using the elastic net [8], since our method identifies cancer biomarkers for each patient rather than the average biomarkers for all samples. However, the drug sensitivity-specific biomarkers discovered by our method were strongly supported as true cancer biomarkers in the literatures (column of “Reference” in Table 6). The result implies that the proposed method for patient-specific analysis produces a reliable result for uncovering cancer biomarkers.

**Table 6:** Identified biomarkers shown the top 10 highest frequency.

Gene	Freq	Reference	Disease
FN1	1,019	[10], [24]	breast cancer, colorectal cancer
TACSTD2	962	[21], [31]	breast cancer
IGL@	960	[15], [27]	metastatic caners, Germ Cell Tumors
IGKCIGKV1-5	957	[12]	leukocytes in human peripheral blood, breast cancer
IGL@IGLC1I	939	[15]	Germ Cell Tumors
COL1A2	935	[17], [21]	breast cancer, prostate cancer
SERPINE2	935	[7]	chronic obstructive pulmonary disease
CD24	855	[16], [33]	Breast Cancer
IFITM3	855	[4], [21]	breast cancer, Colon Cancer
LDHB	833	[23]	Breast Cancer

In short, our method is a useful tool for predicting anti-cancer drug sensitivity and uncovering patient-specific cancer biomarkers.

## Discussion

We have proposed a novel outlier-resistant method for uncovering patient-specific biomarkers and predicting anti-cancer drug response. By using the robust Mahalanobis distance calculated in robust principal component space, the proposed method effectively detects and controls outliers in high dimensional genomic alterations datasets. Thus, the proposed robust method can effectively perform to uncover cancer biomarkers and predict drug sensitivity, even in the presence of outliers. From the Monte Carlo simulations, we have found that our method shows outstanding prediction accuracy as compared to the existing NetworkProfiler and elastic net. We have also applied the proposed method to the Sanger dataset from the Cancer Genome Project. By using our method, we have uncovered cancer biomarkers and predicted anti-cancer drug response. It can be seen from the results that the proposed method is a useful tool for predicting anti-cancer drug response. Furthermore, the biomarkers uncovered by our method had been previously identified as cancer biomarkers. The results implies that our method provides not only reliable feature selection, but also accurate prediction results.

There is currently much discussion about patient-specific analysis and personalized medicine based on high dimensional genomic datasets. We expect that our methodology will be useful for the fields, since genomic data usually contains outliers.

Although the patient-specific method based on a varying coefficient model is an efficient tool, it controls the effects of observations in order to provide sample-specific results. In other words, it reduces the effect of observations far from a target patient, and thus leads to a high dimensional data frame. Building models based on a large number of features with a small number of samples can lead to overfitting in feature selection, and can produce inefficient prediction results. In order to improve modeling performance, future work can involve extending the patient-specific analysis based on the bootstrap technique.

The Sanger dataset from the Cancer Genome Project provides comprehensive information about the molecular features of a cancer (e.g., mutation, expression levels and copy number variation) and response of various anti-cancer drugs. Thus, analysis of the dataset may provide informative results about the systems biology of cancer and valuable information for personalized treatment and anti-cancer therapy. The IC50 values of 138 drugs given as drug sensitivity, however, contain many missing values (from 44 to 364 missing values in total 654 cell lines). In order to effectively use the Sanger dataset to reveal the mechanism of cancer, rather than ignoring the incomplete fields, a proper treatment of the missing values is required.

Furthermore, we have also identified through numerical studies that a large number of noisy features may disturb modeling performance, and thus strategies for pre-selecting a candidate set will be required to improve modeling performance.
